# Depressurization-Induced Nucleation in the “Polylactide-Carbon Dioxide” System: Self-Similarity of the Bubble Embryos Expansion

**DOI:** 10.3390/polym13071115

**Published:** 2021-04-01

**Authors:** Dmitry Zimnyakov, Marina Alonova, Ekaterina Ushakova

**Affiliations:** 1 Physics Department, Yury Gagarin State Technical University of Saratov, 410054 Saratov, Russia; alonova_marina@mail.ru (M.A.); katushakova96@yandex.ru (E.U.); 2 Precision Mechanics and Control Institute of Russian Academy of Sciences, 24 Rabochaya st., 410024 Saratov, Russia

**Keywords:** d,l-polylactide, near-supercritical carbon dioxide, depressurization, bubble embryo expansion, self-similarity

## Abstract

Self-similar expansion of bubble embryos in a plasticized polymer under quasi-isothermal depressurization is examined using the experimental data on expansion rates of embryos in the CO_2_-plasticized d,l-polylactide and modeling the results. The CO_2_ initial pressure varied from 5 to 14 MPa, and the depressurization rate was 5 × 10^−3^ MPa/s. The constant temperature in experiments was in a range from 310 to 338 K. The initial rate of embryos expansion varied from ≈0.1 to ≈10 µm/s, with a decrease in the current external pressure. While modeling, a non-linear behavior of CO_2_ isotherms near the critical point was taken into account. The modeled data agree satisfactorily with the experimental results. The effect of a remarkable increase in the expansion rate at a decreasing external pressure is interpreted in terms of competing effects, including a decrease in the internal pressure, an increase in the polymer viscosity, and an increase in the embryo radius at the time of embryo formation. The vanishing probability of finding the steadily expanding embryos for external pressures around the CO_2_ critical pressure is interpreted in terms of a joint influence of the quasi-adiabatic cooling and high compressibility of CO_2_ in the embryos.

## 1. Introduction

Highly porous bioresorbable polymer matrices are of particular interest as a material platform for creating scaffolds required in regenerative medicine and tissue engineering [1,2,3]. Among the various methods for synthesizing these materials, one of the frequently applied techniques is the depressurization-assisted foaming of pre-plasticized raw polymers in the atmosphere of a supercritical or subcritical plasticizing-foaming agent [4,5,6,7,8,9,10,11,12,13,14,15]. Over the past three decades, a large number of experimental studies on the impact of plasticization and depressurization modes on structural and functional properties of the synthesized polymer matrices as scaffold prototypes have been carried out [6,7,13]. Typically, poly(glycolic acid) PGA, poly(lactic acid) PLA, and their co-polymers were used as raw materials for the synthesis of scaffold prototypes, whereas carbon dioxide was used as a plasticizing/foaming agent. The attractiveness of carbon dioxide as a technological substance is due to a number of factors, such as high solubility in raw polymers, low critical values of temperature and pressure, low cost, a possibility of implementing green technologies, etc.

It should be noted that the field of application of supercritical fluid foaming (SCFF) of polymers is not limited to the synthesis of highly porous biocompatible matrices applied in tissue engineering and regenerative medicine. This technology provides a possibility for engineering not only structural, but also mechanical, electrical, transport and other properties of synthesized porous polymeric materials. Accordingly, we can refer to a number of applied studies carried out in the past decade, which are devoted to the SCFF-assisted synthesis of various functional polymeric materials. In particular, these studies include the design of a versatile foaming platform for the synthesis of composite polymeric materials with a high dielectric permittivity and ultra-low dielectric losses [16,17], creation of ultralight composites for electromagnetic interference shielding [18], the synthesis of microcellular and nanocellular polymeric composites with enhanced mechanical and thermal properties [19,20,21,22], etc.

Formation of a highly porous polymer matrix during depressurization of the “plasticized polymer-carbon dioxide” system includes the following stages:

- Saturation of a raw polymer with a plasticizing/foaming agent by means of required exposure of a processed amount of polymer in an agent atmosphere at a given pressure and temperature;
- Appearance of microscopic CO_2_-filled bubble embryos, randomly distributed in the volume of the plasticized polymer, and evolution of an ensemble of individual bubbles in the polymer volume up to the time of their closure into a foam-like structure during the pressure release;
- Intensive expansion of the polymer foam with a subsequent stabilization of its structure at low pressures.

The dynamics of nucleation and evolution of bubble embryos at the stage preceding the foam expansion has a significant influence on the structural properties of the synthesized matrices. For example, in the case of low nucleation rate and high expansion rate of bubble embryos, a significant variance in the pore sizes of the synthesized matrices should be expected. These rates are controlled by a wide range of variable physical parameters (e.g., viscosity of the plasticized polymer, surface tension of the polymer–carbon dioxide boundaries, solubility, and diffusion coefficient of carbon dioxide in the polymer, etc.) at the current values of temperature and pressure in the “polymer-carbon dioxide” system.

Despite the abundance of experimental and theoretical works devoted to various aspects of nucleation and growth of gas bubbles in such systems, the physical mechanisms controlling the initial bubbling stage in the plasticized polymers are still far from fully understood. In particular, this is due to the complexity of accompanying physical processes, and peculiarity in the behavior of carbon dioxide as a foaming agent in the range of pressures and temperatures near the critical point. This peculiarity is related to significant changes in the density at small pressure variations.

Analysis of a significant amount of empirical data on depressurization-assisted synthesis of highly porous polymer matrices [23,24,25,26] allows us to conclude that the maximum expansion factor Θ of initially plasticized raw polymers occurs within a relatively narrow range of initial pressures $P_{ext}^{in}$ of the plasticizing/foaming agent. The expansion factor is defined as the ratio of the synthesized foam volume to the initial volume of a plasticized polymer. In particular, the maximum values of Θ in the course of foaming the amorphous d,l-polylactide plasticized in the carbon dioxide atmosphere are achieved in the range of 8.0 MPa $\leq P_{ext}^{in}\leq$ 12 MPa around the critical pressure of $P_{c}\approx$ 7.377 MPa of CO_2_ [26]. 

**Figure 1:**
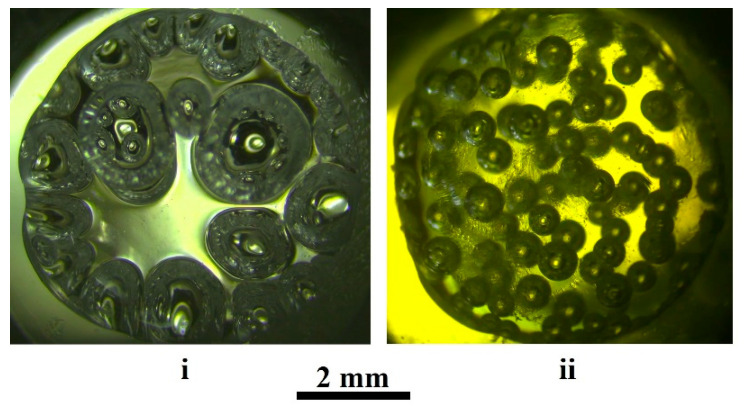
Samples of the quasi-isothermally foamed d,l-polylactide at the beginning of an intensive foam expansion. i—$P_{ext}^{in}=$ 6.0 MPa, T= 323 K; ii—$P_{ext}^{in}=$ 11.5 MPa, T= 323 K.

Compared with the cases of significant detuning of the initial pressure from the critical value ($P_{ext}^{in}\leq$ 6.0 MPa or $P_{ext}^{in}\geq$ 12.0 MPa), the polylactide matrices synthesized under these conditions are characterized by a significantly more homogeneous structure. As an illustration of this feature, Figure 1 shows the images of polylactide foams at the initial stage of intensive expansion in the course of slow quasi-isothermal depressurization. Case (i) corresponds to a remarkable detuning of the initial pressure below the critical value, while case (ii) corresponds to the foaming mode close to the optimal conditions. Intuitively, we can assume that to obtain the foamed matrices with a more homogeneous structure, it is advisable to use the foaming modes with higher nucleation rates but with low or medium expansion rates of the formed bubble embryos. Indeed, if the nucleation rate is low, then the number of embryos nucleated during a short time interval will be small. With high expansion rates of evolving embryos, the differences in the size of the bubbles appearing at different times will be large, and this will lead to significant variability in the bubble sizes.

Highly porous polymer matrices synthesized using the technology discussed (depressurization-assisted foaming in the atmosphere of supercritical/subcritical blowing agent) are characterized, depending on the applied depressurization conditions, with an average pore size ranging from tens of micrometers to a few millimeters and a significant scattering of pore sizes. In particular, examination of the structures of polylactide matrices synthesized in a similar way using micro-X-ray computed tomography and scanning electron microscopy [14] have shown a significant effect of the depressurization rate on the average pore size. In this study, d,l-polylactide specimens with different molecular weights were examined, and initial values of the CO_2_ pressure and temperature were chosen equal to 23.2 MPa and 308.15 K. The depressurization rate varied from ≈3.9 × 10^−2^ MPa/s to ≈6.5 × 10^−3^ MPa/s. The synthesized matrices [14] are characterized by the values of porosity varying insignificantly around ≈80%, depending on the molecular weight of specimens and the applied depressurization rate. In the case of d,l-polylactide samples with the minimal molecular weight (15 kDa), the average size of pores systematically rises from ≈300 to ≈800 µm when the depressurization rate increases. On the contrary, the specimens with higher molecular weights (24 and 57 kDa) demonstrate a significantly weaker effect of the depressurization rate on the average size of pores. A decrease in the depressurization rate also leads to broadening the pore size distributions; this trend was observed in the experiments on foaming other biocompatible polymers (amorphous PLGA [7] and a highly crystalline random co-polymer of x-pentadecalactone (PDL) and e-caprolactone (CL) (poly(PDL-CL)) [27]).

In addition to the rate of pressure release, the initial parameters (pressure and temperature) have a significant effect on the final structure of the formed matrices. As mentioned above, Figure 1 illustrates a dramatic increase in the average bubble size and width of their size distribution at the beginning of intensive foam expansion with a decrease in the initial pressure under conditions of a constant release rate and temperature. At the final stage of foaming, a highly porous matrix (case “i”) is characterized by an average size of pores of the order of few millimeters, and an extremely large scatter of pore sizes compared to “ii”.

Accordingly, it should be noted that despite the abundance of theoretical and experimental works devoted to various aspects of the formation and development of ensembles of gas bubbles in metastable two-phase systems, so far, the influence of various factors on the dynamics of these processes in such complex systems as expandable polymers has not been fully investigated. “Polymer-plasticizing/foaming agent” systems under depressurization are a particular case of these two-phase systems. The purpose of this work is an experimental study, for modeling of the features associated with the bubble embryo development in the plasticized polylactide during a slow quasi-isothermal depressurization at the stage preceding an intensive expansion of the foam (the pre-foaming stage). In addition to the applied aspects related to the synthesis of highly porous bioresorbable matrices as a material platform for scaffolds, this study seems to be useful in terms of a better understanding of physical processes in the systems “polymer-plasticizing/foaming agent” during the transitions between different structural states. In the framework of this study, analysis of the influence of external conditions on the dynamics of bubble embryo evolution in the “d,l-polylactide-carbon dioxide” system during a quasi-isothermal depressurization was carried out. Additionally, the physical mechanisms controlling expansion of individual bubble embryos in the plasticized polymer are considered in the framework of the developed phenomenological model.

## 2. Materials and Methods

The experimental data on the expansion dynamics for individual bubble embryos in the course of slow quasi-isothermal depressurization of the system “polylactide-carbon dioxide” were obtained using the materials, techniques, and instrumentation similar to those described elsewhere [26].

### 2.1. Materials

In the course of experimental studies, the amorphous d,l-polylactide (PURASORB PDL 04, CAS number 26680-10-4, product #1824008 of Corbion Purac, Amsterdam, The Netherlands) was used as a plasticized/foamed material. PURASORB PDL 04 is a GMP (Good Manufacturing Practice) grade copolymer of d,l-lactide with an inherent viscosity midpoint of 0.4 dl/g. It is supplied in granular form and primarily used in biomedicine and drug delivery. d,l-polylactides are usually considered as almost fully amorphous polymers; therefore, the glass transition temperature is much more appropriate for characterization of the examined samples than the melting temperature. According to the previously provided DSC (differential scanning calorimetry) measurements using DSC 214 Polyma^®^ system (NETZSCH, Selb, Germany), this raw material is characterized by the glass transition temperature approximately equal to 320.15 K [26]. Chemically pure carbon dioxide (p.a. grade, product of Cryogen company, Balashikha, Russia) was applied as a plasticizing/foaming agent.

### 2.2. Methods

**Figure 2:**
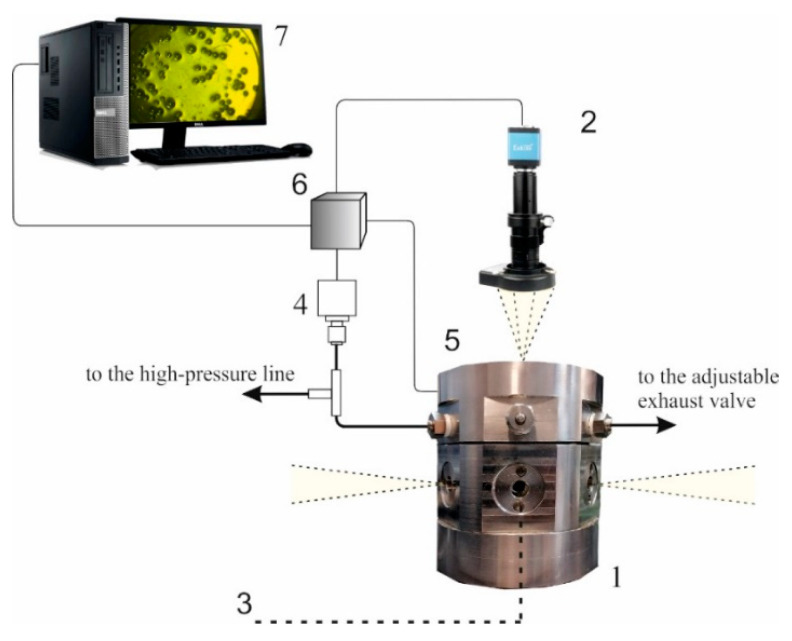
Experimental equipment for the analysis of bubbling in carbon dioxide-plasticized D,L-polylactide. 1—multi-window high-pressure chamber; 2—CMOS camera with the macro-lens and illumination assembly; 3—sapphire glass window (one of seven); 4—pressure gauge; 5—output of the thermocouple; 6—data acquisition assembly; 7—PC. Resistance-type heaters are not shown. Yellow-filled sectors display the side-window and upper-window illumination. The chamber is connected with the CO_2_-filled controllable high-pressure line via a shutoff valve (not shown).

Portions of 20 mg preliminary criomilled polylactide (the average size of grains is in the order of 100 micrometers) were plasticized in the carbon dioxide environment under fixed conditions (carbon dioxide pressure and temperature) inside a multi-window high-pressure chamber (Figure 2). The samples of the polylactide powder in the open cylindrical glass cells with flat bottom walls were exposed for one hour under the given pressure of the chamber-filling carbon dioxide (in the range from 5.0 to 14.0 MPa) at the fixed temperature (in the range from 310 to 338 K). The current values of pressure and temperature inside the thermally stabilized high-pressure chamber were constantly measured with random errors no worse than ±0.02 MPa and ±0.05 K. The data flows from the preliminary calibrated thermocouple (K type) and precision pressure sensor (APZ3421, PIEZUS, Moscow, Russia) were digitized using a 14-bit format and recorded at a 30 Hz sampling rate. In each experiment, at the end of the plasticization exposure, homogeneous solutions of carbon dioxide in plasticized polylactide filled cuvettes were produced. Concentrations of carbon dioxide in the plasticized polylactide depended on the CO_2_ solubility at the given temperature and pressure. Controllable quasi-isothermal depressurization of the examined systems was provided with the pressure drop rate approximately equal to $dP_{ext}/dt\approx$ 5 × 10^−3^ MPa/s using an automatically adjusted exhaust needle valve. The valve was heated up to 40 °C to exclude its freezing under depressurization. Recordings of the current temperature inside the chamber during the given slow pressure decrease showed that the temperature variations under these depressurization conditions did not exceed ±0.2 K along the whole depressurization interval. Therefore, we can identify this pressure drop mode as quasi-isothermal depressurization.

Over the course of depressurization, the sequences of images of the plasticized polylactide samples were captured through the upper window of a high-pressure chamber using an assembly consisting of a CMOS camera XCAM1080PHB (ToupTec, Hangzhou, China) and macro-lens with an adjustable magnification; the frame rate during the image capturing and recording was equal to 30 fps. The cuvettes with the examined samples were illuminated by a diffuse light through the upper and side windows of the chamber. The camera lens was adjusted in such a way that the object plane was buried to the polymer layer by approximately half of its thickness (≈0.5 mm). This made it possible, to a certain extent, to minimize the errors in evaluating the size of embryos that appear at different depths in the polymer layer. At the same time, it should be noted that the depth of the field of the imaging system is large enough to make defocus distortions small enough.

The image recordings were synchronized with the recordings of data flows from the temperature and pressure sensors. Resolution of the captured images was equal to 1920 × 1080 pixels; with the lateral magnification of the macro-lens, it corresponded to a scaling factor of 4.2 micrometers/pixel. 

**Figure 3:**
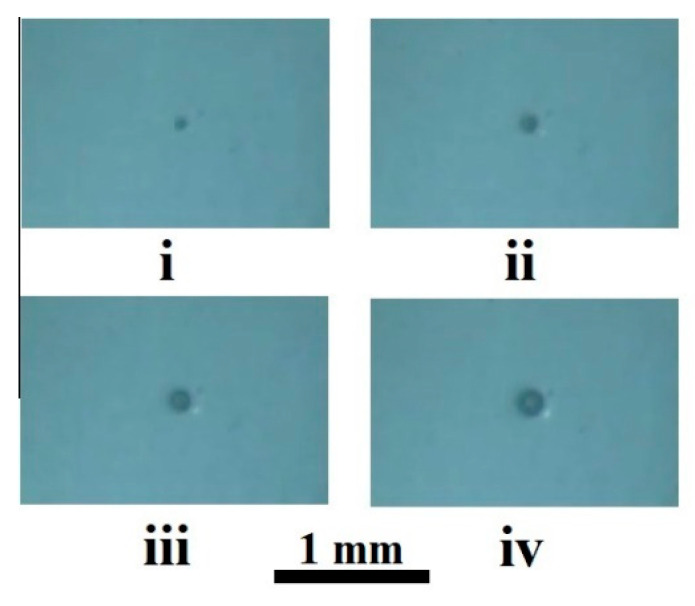
Sizing of an individual embryo in the course of depressurization. The initial conditions are: $P_{ext}^{in}=$ 11.5 MPa, T= 310 K. The values of ${\bar{P}}_{ext}$ are: i—5.86 MPa; ii—5.75 MPa; iii—5.71 MPa; iv—5.65 MPa.

In the course of image analysis, depressurization-induced evolution of the single bubble embryos randomly appearing in initially homogeneous plasticized polylactide was studied depending on the current external pressure $P_{ext}$ in the chamber. In general, the time intervals applied for the analysis in each experiment were limited by the initial formation stage of an ensemble of bubbles at a given temperature and rate of pressure release. Regarding the current values of the number of bubble embryos N(t) in the field of the view of the camera and the average radius of the embryo images 〈R(t)〉, these time intervals are limited by the following condition: $\sqrt{S/N\left(t\right)}>>\langle R\left(t\right)\rangle$, where S is the area of the region of interest. Figure 3 illustrates an increase in the current radii of an individual embryo chosen for the analysis with a decreasing external pressure.

Analysis of the embryo image sequences was conducted in the interactive mode using specially developed MATLAB software. In the framework of this procedure, a new embryo that appeared in the area of interest at some point in time was selected in the manual mode and identified by the coordinates in the image plane. The selected embryo was traced along the image sequence with evaluation of the image radius at each step in the sequence as $R\left(t\right)\approx \sqrt{\Delta S\cdot N\left(t\right)/\pi }$, where N(t) is the number of pixels covered by the chosen embryo image at the moment, and ΔS is the area corresponding to a single pixel. The obtained time series R(t) was smoothed using a running Gaussian window with a half-width equal to a ten-fold sampling interval of image acquisition (≈0.33 s). A similar procedure was applied to the corresponding sequence of pressure values $P_{ext}\left(t\right)$. As a result, the dependencies $\bar{R}\left({\bar{P}}_{ext}\right)$ corresponding to the appearing and expanding individual embryos were recovered for the given depressurization experiment carried out at the given temperature. These dependencies were used to evaluate the embryo expansion rates $\left|{\Delta \bar{R}/\Delta {\bar{P}}_{ext}|}_{\bar{R}\to R_{c}}\right|$ at the beginning of expansion by applying the following procedure. For each sequence of the smoothed data $\bar{R}\left({\bar{P}}_{ext}\right)$, a constant value of the external pressure decrement $\Delta {\bar{P}}_{ext}={\bar{P}}_{ext,i}-{\bar{P}}_{ext,1}$was set equal to$0.001{\bar{P}}_{ext,0}$ and a corresponding increment of the embryo radius $\Delta \bar{R}={\bar{R}}_{i}-{\bar{R}}_{1}$ was evaluated. Here, ${\bar{P}}_{ext,0}$ is an extrapolated value of the external pressure for the moment of embryo appearance, the subscript “1” corresponds to the first term in the data sequence, and the ith term corresponds to the external pressure equal to $\approx 0.999{\bar{P}}_{ext,0}$. The notation $R_{c}$ stands for an unknown initial radius of a bubble embryo.

**Figure 4:**
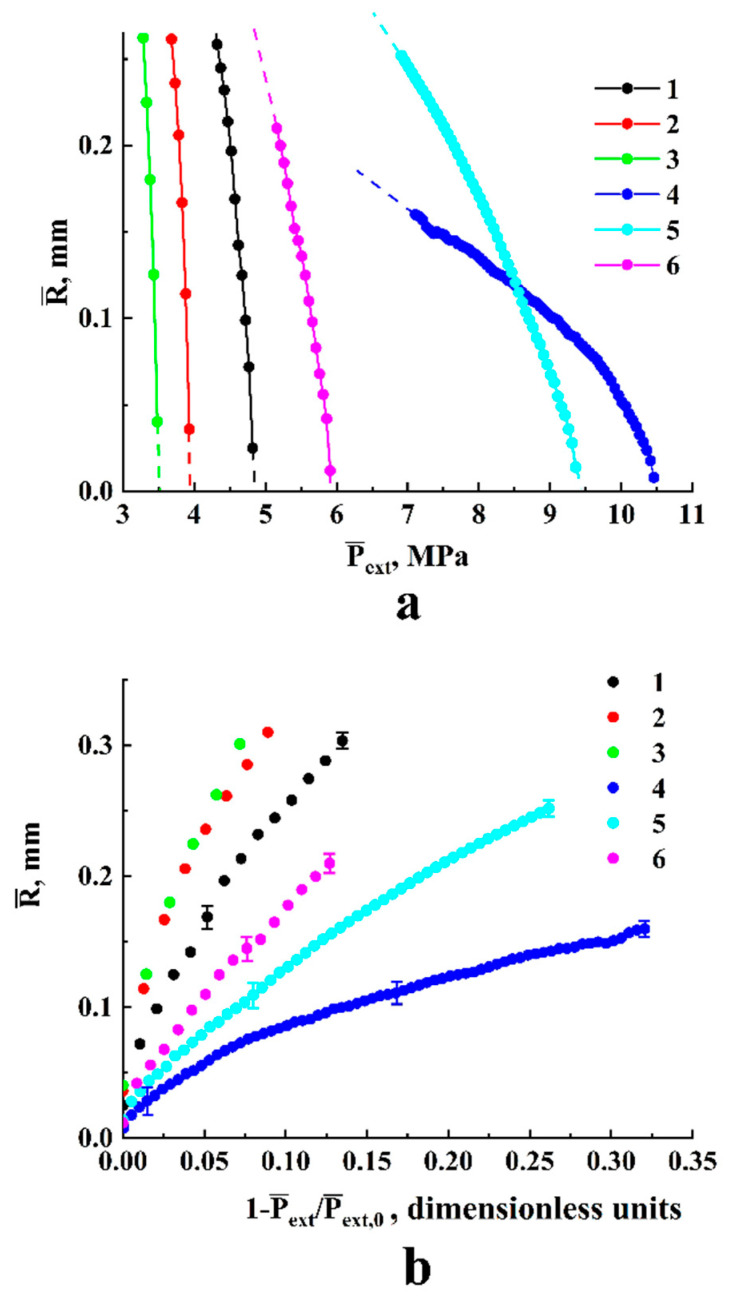
Depressurization-driven expansion of bubble embryos depending on the temperature and initial pressure. Smoothed current radii of expanding embryos are displayed against the current values of ${\bar{P}}_{ext}$ (**a**) and $1-{\bar{P}}_{ext}/{\bar{P}}_{ext,0}$ (**b**).

**Figure 5:**
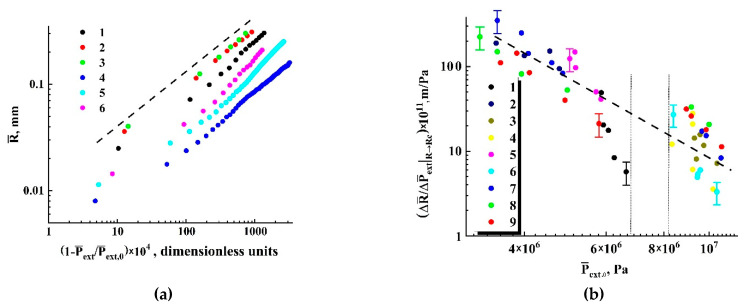
(**a**) Smoothed radii of embryos against $1-{\bar{P}}_{ext}/{\bar{P}}_{ext,0}$ in logarithmic coordinates. Assignment of the markers is the same as in Figure 4. The dashed line marks $\bar{R}\propto {\left(1-{\bar{P}}_{ext}/{\bar{P}}_{ext,0}\right)}^{0.5}$ trends in the expansion. (**b**) The estimated values of $\left|{\Delta \bar{R}/\Delta {\bar{P}}_{ext}|}_{R\to R_{c}}\right|$ against ${\bar{P}}_{ext,0}$. The dashed line marks the observed trend $\left|{\Delta \bar{R}/\Delta {\bar{P}}_{ext}|}_{R\to R_{c}}\right|\propto {\left({\bar{P}}_{ext,0}\right)}^{-2.7}$.

Figure 4 and Figure 5 display examples of the recovered data for various experimental conditions. A remarkable feature is a significant increase in the initial expansion rate $\left|{d\bar{R}/d{\bar{P}}_{ext}|}_{\bar{R}\to R_{c}}\right|$ with the decreasing external pressure $P_{ext}$. The error bars selectively shown in Figure 4b and Figure 5b correspond to the confidence level of 0.9 and cover the expected random deviations due to various uncertainties. The applied experimental conditions are given in Table 1 and Table 2. Figure 5a illustrates the general trend in the behavior of expanding individual embryos with the increasing detuning factor $\xi =1-{\bar{P}}_{ext}/{\bar{P}}_{ext,0}$ (here, the extrapolated values ${\bar{P}}_{ext,0}$ correspond to the condition $\bar{R}\to R_{c}$). This trend is correlated with the approximating power-law function $R\propto \xi^{0.5}$ for all the traced individual embryos if ξ exceeds ≈0.001.

Note that the displayed remarkable scattering of the data recovered for the close values of ${\bar{P}}_{ext,0}$ results not only from relatively large recovery errors for the small embryos (see the error bars in Figure 4b), but also from stochasticity of bubble nucleation. Nucleating bubble embryos able to further stably expand are characterized by a certain probability distribution of $R_{c}$ depending on the current external conditions (${\bar{P}}_{ext}$ and T). In turn, as shown below, the $R_{c}$ value affects the rate of its further expansion. Despite a significant scattering of the recovered values $\left|{d\bar{R}/d{\bar{P}}_{ext}|}_{\bar{R}\to R_{c}}\right|$, the general trend in their behavior clearly manifests itself in Figure 5b. This trend can be specified with a reasonable accuracy by the power-law function $\left|{d\bar{R}/d{\bar{P}}_{ext}|}_{\bar{R}\to R_{c}}\right|\propto {\bar{P}}_{ext}^{\delta }$ with the exponent δ equal to about 2.7.

Another important feature of the diagram displaying $\left|{d\bar{R}/d{\bar{P}}_{ext}|}_{\bar{R}\to R_{c}}\right|$ against ${\bar{P}}_{ext}$ is occurrence of a gap zone corresponding to the absence of experimentally fixed expanding embryos around the value of critical pressure of carbon dioxide (this gap zone is marked by the vertical dotted lines in Figure 5b). Presumable reasons for these features are discussed below.

## 3. Modeling of Individual Bubble Expansion in the Depressurized System “Polylactide-Carbon Dioxide”

### 3.1. The Applied Kinetic Model

We consider the following concept in the description of the behavior of plasticized polymer at the depressurization stage preceding a close packing of individual bubbles and intensive expansion of the polymer foam: 

(1) With a gradual decrease in external pressure, solution of carbon dioxide in the polylactide becomes a thermodynamically metastable two-phase system, where the nucleation rate of the second phase (carbon dioxide in the supercritical or subcritical state) at the current pressure becomes remarkably different from zero;

(2) With a certain probability, some of the appearing nuclei (bubble embryos) are characterized by the radius R equal to or exceeding the critical radius $R_{c}=2\sigma /\left(P_{\mathrm{int}}-P_{ext}\right)$ for the current depressurization conditions; this part forms a random ensemble of steadily expanding non-interacting bubble embryos with an ever-increasing number of ensemble members; other embryos with $R<R_{c}$ are instable and collapse over time; here, $P_{\mathrm{int}}$ is the current internal pressure of carbon dioxide in the embryo, and σ is the current surface tension of the embryo boundary; 

(3) Within the framework of the model, evolution of an arbitrarily chosen expanding individual embryo is analyzed for a relatively small range of the detuning factor 0.001$\leq \xi =1-P_{ext}/P_{ext,0}\leq$ 0.1;

(4) Parameters of the “polymer-carbon dioxide” system vary insignificantly during the initial expansion stage of a given individual bubble (see point 3); therefore, we assume the surface tension, polymer viscosity, CO_2_ diffusion coefficient, etc., as constant values for the given expansion act, but varying from one expansion act to another depending on the temperature and external pressure at the time of embryo formation; this assumption can be accepted if the maximum value of the detuning parameter ξ in the expansion analysis is significantly less than 1;

(5) Interactions of the given bubble embryo with the neighboring embryos and the surface of the plasticized polymer are negligible. 

It is necessary to note a rather large number of theoretical studies devoted to the analysis of stability conditions and expansion dynamics of the isolated gas bubbles in supersaturated solutions, which have been carried out since the 1950s [28,29,30,31,32]. However, a vast majority of these studies are limited to the assumption that the substance filling the bubble behaves as an ideal gas described by the general gas equation $P_{\mathrm{int}}=\rho_{\mathrm{int}}\tilde{R}T$ (here, $P_{\mathrm{int}},\rho_{\mathrm{int}}$ are the pressure and density of the substance in the bubble, $\tilde{R}$ is the specific (molar) gas constant, and T is the absolute temperature). In the vicinity of the critical point of the solute, the relationship between internal pressure and density becomes substantially nonlinear, and this should be taken into account in the further analysis.

The original equation describing the dynamics of expansion of a single bubble in a viscous infinite medium (the so-called modified Rayleigh equation) is written as [30]:

(1) $$\frac{4\eta }{R}\cdot \frac{dR}{dt}+R\rho_{p}\frac{d^{2}R}{dt^{2}}+\frac{3\rho_{p}}{2}{\left(\frac{dR}{dt}\right)}^{2}=P_{\mathrm{int}}-P_{ext}-\frac{2\sigma }{R},$$

where η and $\rho_{p}$ are the dynamic viscosity (Pa·s) and the density of the surrounding medium. We can neglect the terms $R\rho_{p}\left(d^{2}R/dt^{2}\right)$ and $\rho_{p}{\left(dR/dt\right)}^{2}$ on the left-hand side of Equation (1) due to the experimental conditions (low depressurization rates causing the expansion rates in the range from ≈0.1 to ≈10 µm/s). Correspondingly, we reduce Equation (1) to the following form:

(2) $$\frac{dR}{dt}\approx \frac{R}{4\eta }\cdot \left(P_{\mathrm{int}}-P_{ext}-\frac{2\sigma }{R}\right).$$

To complete the description of the system, we must additionally introduce the mass balance equation in the following form:

(3) $$\frac{d}{dt}\left(\frac{4\pi }{3}R^{3}\rho_{\mathrm{int}}\right)={4\pi R^{2}\frac{\mu }{N_{A}}D\frac{\partial c}{\partial r}|}_{r=R},$$

The equation of state of the substance (in our case, carbon dioxide) in an expanding bubble must be introduced as well:

(4) $$\rho_{\mathrm{int}}=f_{T}\left(P_{\mathrm{int}}\right).$$

Here, D is the diffusion coefficient of the solute in the plasticized polymer, μ is the molecular mass of the solute, $N_{A}$ is the Avogadro number, and c(r) describes the radial distribution of solute concentration in the plasticized polymer around the bubble. Equation (3) describes the diffusion transfer of the solute from the surrounding zone of the plasticized polymer to the bubble embryo with the transfer rate proportional to the concentration gradient at the bubble–polymer interface. For a further analysis, we can assume that the diffusion fluence rate $\left(\mu /N_{A}\right)D\left({\partial c/\partial r|}_{r=R}\right)$ at a given time can be considered as the function $\varphi \left(R,\rho_{\mathrm{int}}\right)$ of the current bubble radius and the solute density in the bubble. An approximate form of $\varphi \left(R,\rho_{\mathrm{int}}\right)$ is discussed below.

Equation (3) can be transformed to the following form:

(5) $$\frac{d\rho_{\mathrm{int}}}{dt}=\frac{3}{R}\left\{{\frac{\mu }{N_{A}}D\frac{\partial c}{\partial r}|}_{r=R}-\frac{dR}{dt}\rho_{\mathrm{int}}\right\}.$$

The relationship between $\rho_{\mathrm{int}}$ and $P_{\mathrm{int}}$ is determined by Equation (4) of the state of the solute as a single-component system, which can be considered in the numerical form.

Earlier, several attempts were made to describe the radial distribution $c{\left(r\right)}_{r\geq R}$ of the solute concentration in the region around the isolated bubble [33,34,35,36]. All these attempts are based on the assumption that there is a finite cutoff distance R+δ from the center of the bubble, for which the condition c(R+δ)=c(∞) is satisfied (here, c(∞) is the solute concentration in the undisturbed solution far away from the bubble). In particular, Han and Yoo [34] assumed a simple parabolic concentration profile:

(6) $$\frac{c\left(\infty \right)-c\left(r\right)}{c\left(\infty \right)-c\left(R\right)}={\left(1-\frac{r-R}{\delta }\right)}^{2}.$$

Payvar [35] considered a slightly more complicated form of the radial distribution:

(7) $$\frac{c\left(\infty \right)-c\left(r\right)}{c\left(\infty \right)-c\left(R\right)}=\frac{r}{R}{\left(1-\frac{r-R}{\delta }\right)}^{2}.$$

Shafi et al. [36] proposed a cubic form for the concentration profile:

(8) $$\frac{c\left(\infty \right)-c\left(r\right)}{c\left(\infty \right)-c\left(R\right)}=\frac{r^{3}-R^{3}}{{\left(R+\delta \right)}^{3}-R^{3}}.$$

All the models clearly predict an approximately inverse proportionality between the concentration gradient at the bubble boundary and the cutoff distance:

(9) $${\frac{\partial c}{dr}|}_{r=R}\sim \frac{\left\{c\left(\infty \right)-c\left(R\right)\right\}}{\delta }$$

In the further analysis, we introduce the following assumptions:

- The concentration profile c(r) around a slowly expanding bubble embryo can be considered as a self-similar solution of a non-stationary diffusion equation, and the current cutoff radius δ is related to the current bubble radius as δ∝R;
- The monotonically decaying term $\left(\mu /N_{A}\right)\cdot \left\{c\left(\infty \right)-c\left(R\right)\right\}$ is considered as an expansion in the series of the powers of density $\rho_{\mathrm{int}}$, with only the first (linear) term taken into account; consequently, this term is assumed proportional to $\rho_{\mathrm{int}}$ with a small proportionality coefficient: $\left(\mu /N_{A}\right)\cdot \left\{c\left(\infty \right)-c\left(R\right)\right\}<<\rho_{\mathrm{int}}$.

With these assumptions, Equation (5) can be rewritten into the following form:

(10) $$\frac{d\rho_{\mathrm{int}}}{dt}=\frac{3\rho_{\mathrm{int}}}{R}\left\{\frac{KD}{R}-\frac{dR}{dt}\right\}.$$

Here, K is a dimensionless phenomenological parameter taking into account the assumed relationship between δ and R, as well as between $\left(\mu /N_{A}\right)\cdot \left\{c\left(\infty \right)-c\left(R\right)\right\}$ and $\rho_{\mathrm{int}}$.

Introducing a set of dimensionless parameters, we arrive to the system of dimensionless equations describing the expansion dynamics of a single embryo filled by the solute (carbon dioxide):

(11) $$\left\{ \begin{array}{l} \frac{d\tilde{R}}{d\tau }=\frac{\tilde{R}}{4}\left({\tilde{P}}_{\mathrm{int}}-{\tilde{P}}_{ext}-\frac{\Sigma }{\tilde{R}}\right),\\ \frac{d{\tilde{\rho }}_{\mathrm{int}}}{d\tau }=\frac{3{\tilde{\rho }}_{\mathrm{int}}}{\tilde{R}}\left(\frac{\Xi }{\tilde{R}}-\frac{d\tilde{R}}{d\tau }\right),\\ {\tilde{\rho }}_{\mathrm{int}}=\varphi_{T}\left({\tilde{P}}_{\mathrm{int}}\right), \end{array}\right.$$

These equations have the following initial conditions: $\tilde{R}\left(0\right)=1;{\tilde{\rho }}_{\mathrm{int}}\left(0\right)=1;{\tilde{P}}_{\mathrm{int}}\left(0\right)=1$. The dimensionless time τ is introduced as $\tau =\left(P_{\mathrm{int},0}/\eta \right)\cdot t$, where $P_{\mathrm{int},0}$ is an internal pressure in the bubble embryo with the critical radius $R_{c}$, which can steadily expand. Accordingly, τ=0 corresponds to the moment of formation of a steadily expanding bubble, $\tilde{R}=R/R_{c}$, ${\tilde{P}}_{\mathrm{int}}=P_{\mathrm{int}}/P_{\mathrm{int},0}$, ${\tilde{P}}_{ext}=P_{ext}/P_{ext,0}$ ${\tilde{\rho }}_{\mathrm{int}}=\rho_{\mathrm{int}}/\rho_{\mathrm{int},0}.$ Dimensionless parameters Σ and Ξ are introduced as follows:

(12) $$\left\{ \begin{array}{l} \Sigma =\frac{2\sigma }{R_{c}P_{\mathrm{int},0}},\\ \Xi =\frac{\eta }{P_{\mathrm{int},0}}\cdot \frac{KD}{R_{c}^{2}}. \end{array}\right.$$

It is useful to discuss the physical meaning of these dimensionless factors; the first characterizes the ratio of excess Laplace pressure associated with the curvature of the bubble embryo boundary to the internal pressure related to the density of the precipitated solute in subcritical or supercritical state. The second factor, Ξ, characterizes the relationship between the rates of the density increase due to solute diffusion into the bubble and decrease due to bubble expansion. It is obvious that the necessary condition for a stable growth of incipient bubble embryos is a positive value of the extra-pressure $\Delta P=P_{\mathrm{int}}-P_{ext}-2\sigma /R$ inside the embryo. Thus, within the framework of the considered model, the viscosity η of the surrounding polymer affects the kinetics of the expanding nucleus in two ways: through the characteristic time $\eta /P_{\mathrm{int},0}$ and through the generalized parameter Ξ.

In terms of modeling, it is important to define the expected ranges of model parameters for the examined system under the experimental conditions. In particular, the expectable range of the normalized extra-pressure $\tilde{\Delta P_{\tau =0}}=\Delta P\left(t=0\right)/P_{\mathrm{int},0}$ at the initial stage of expansion can be roughly estimated using the values of expansion rates and expected bubble sizes (of the order of a few micrometers), and the reported data on dynamic viscosity of the CO_2_-plasticized amorphous polylactide (of the order of several hundred Pa·s [37,38]. In our case, the estimated values of dR/dt vary from 0.1 µm/s to 10 µm/s depending on initial depressurization conditions. Accordingly, the normalized initial extra-pressure for a steadily expanding bubble embryo must not exceed ≈10^−3^. Regarding the term Σ, we can roughly evaluate this parameter using the reported data on the surface tension of the plasticized polylactide [39] (σ≈ (3÷6) × 10^−2^ N/m), and expected values of the initial radii $R_{c}$. Consequently, the normalized initial Laplace pressure Σ is expected around ≈10^−2^.

**Figure 6:**
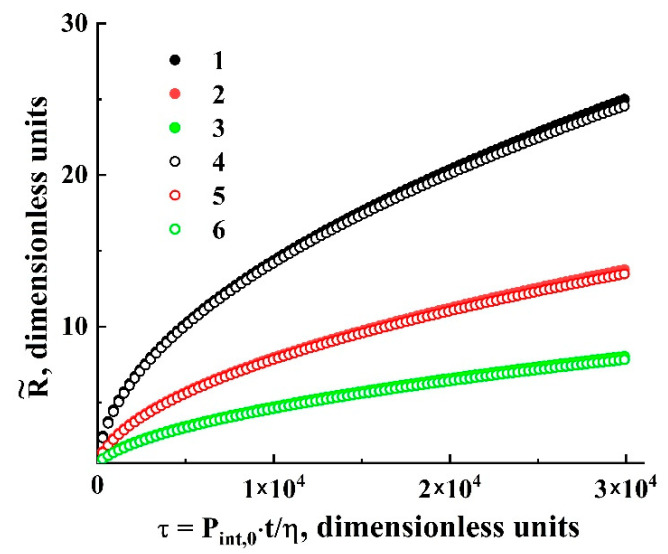
Theoretical dependencies of $\tilde{R}$ on the dimensionless time lapse τ. 1,4—Ξ= 0.01; 2,5—Ξ=0.003; 3,6—Ξ= 0.001. The datasets (1–3) correspond to T= 310 K and $P_{\mathrm{int},0}=$ 8.3 MPa; the datasets (4–6) correspond to T= 333 K and $P_{\mathrm{int},0}=$ 10.0 MPa.

The system of Equation (11) was solved using the finite difference technique; the normalized equation of state of the solute (carbon dioxide) in the expanding bubble at a given constant temperature was recovered in the numerical form using isothermal datasets (the density against the pressure). These datasets for various temperatures applied in the experiments were generated using the online NIST calculator of thermophysical properties of fluid systems [40]. Figure 6 displays the family of theoretical dependencies $R/R_{c}=f\left(P_{\mathrm{int},0}t/\eta \right)$ calculated in a wide range of Ξ (from 0.001 to 0.01). Comparing the trends in the behavior of the model system with experimentally observed expansion dynamics of the single bubbles in the plasticized polylactide, we can emphasize an essential role of diffusion transport of the solute in the growing bubbles described by Ξ, in the course of slow depressurization. In particular, the characteristic values of Ξ for the examined system “polylactide-carbon dioxide” are expected in the range from 0.001 to 0.005.

### 3.2. Self-Similarity of Bubble Expansion

**Figure 7:**
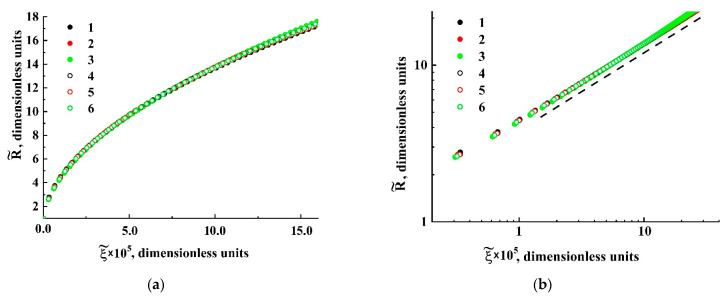
The result of transformation of the modeled dependencies $\tilde{R}=\psi \left\{\tau \right\}$ to the dependence $\tilde{R}=\psi \left\{\tilde{\xi }\right\}$ in the linear (**a**) and logarithmic (**b**) coordinates. The assignment of markers is the same as in Figure 6. The dotted line in (**b**) serves as a guide for the eye and corresponds to $\tilde{R}\propto {\tilde{\xi }}^{0.5}$.

The main feature of the modeled bubble expansion is self-similarity of the dependences $\tilde{R}=\psi \left\{\xi \right\}$ in the region of relatively small values of the detuning parameter $\xi =1-{\tilde{P}}_{ext}/{{\tilde{P}}_{ext}|}_{R\to R_{c}}$ (1.0 × 10^−4^≤ξ≤0.02) for a wide range of the dimensionless parameter Ξ. The obtained model dependences $\tilde{R}\left(\tau \right)$ characterized by a significant divergence with an increasing dimensionless time lapse τ (Figure 6) can be transformed to the unique function $\tilde{R}=\psi \left\{\tilde{\xi }\right\}$ (Figure 7a) using a change in the variable τ→ξ and the renormalization procedure $\Xi \cdot \xi \to \tilde{\xi }$. Even in spite of significant discrepancies in the behavior of normalized isotherms of carbon dioxide at different values of the initial pressure and temperature detuning from the critical value, the bubble expansion can be adequately considered as a self-similar process, even for relatively large values of ξ

**Figure 8:**
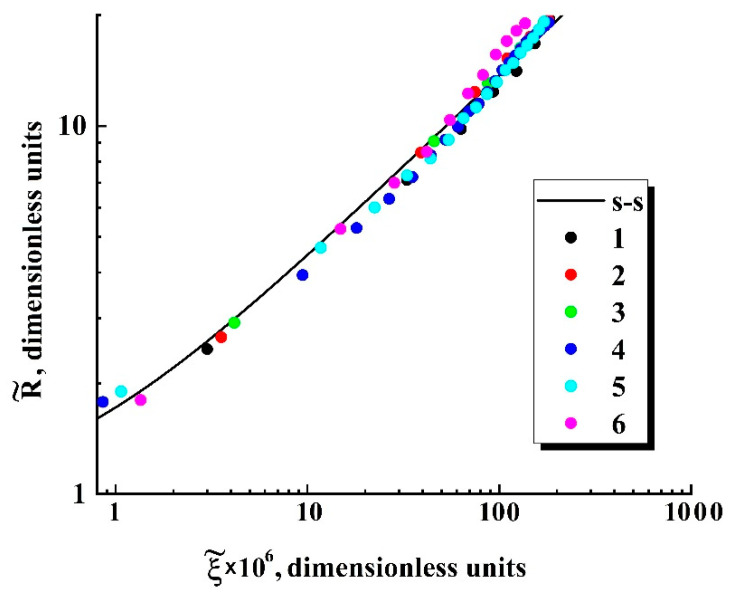
Fitting of the empirical data (Figure 5a) based on the self-similar dependence (s-s) $\tilde{R}=\psi \left\{\tilde{\xi }\right\}$(Figure 7). The assignment of the markers (1–6) corresponds to that accepted for Figure 4 and Figure 5a.

Two characteristic stages can be identified in the behavior of the function $\tilde{R}=\psi \left\{\tilde{\xi }\right\}$(Figure 7b): a transient stage in the region of small $\tilde{\xi }$ ($\tilde{\xi }\leq$ 3 × 10^−4^) and self-similar power-law growth $\psi \propto {\tilde{\xi }}^{0.5}$ with the exponent α= 0.5 as the dimensionless variable $\tilde{\xi }$ further increases. Note that the latter feature qualitatively agrees with the abovementioned trend in the behavior of obtained empirical dependencies of the smoothed bubble radii $\bar{R}$ on the detuning parameter $1-{\bar{P}}_{ext}/{{\bar{P}}_{ext}|}_{\bar{R}\to R_{c}}$ (Figure 5a). Accordingly, these empirical data can be fitted to the theoretically obtained self-similar function $\tilde{R}=\psi \left\{\tilde{\xi }\right\}$ using the appropriately chosen parameters $R_{c}$ and Ξ; Figure 8 displays the result of this fitting procedure; the fitting parameters for each dataset are given in Table 3. Analyzing the behavior of the initial embryo radius $R_{c}$ as the fitting parameter for the experimental dataset, we can see that it systematically increases with the decreasing external pressure $P_{ext,0}$ corresponding to the initiation of embryo expansion. A presumable reason for such behavior is discussed in the following section.

### 3.3. A Transient Mode of the Bubble Embryo Expansion 

**Figure 9:**
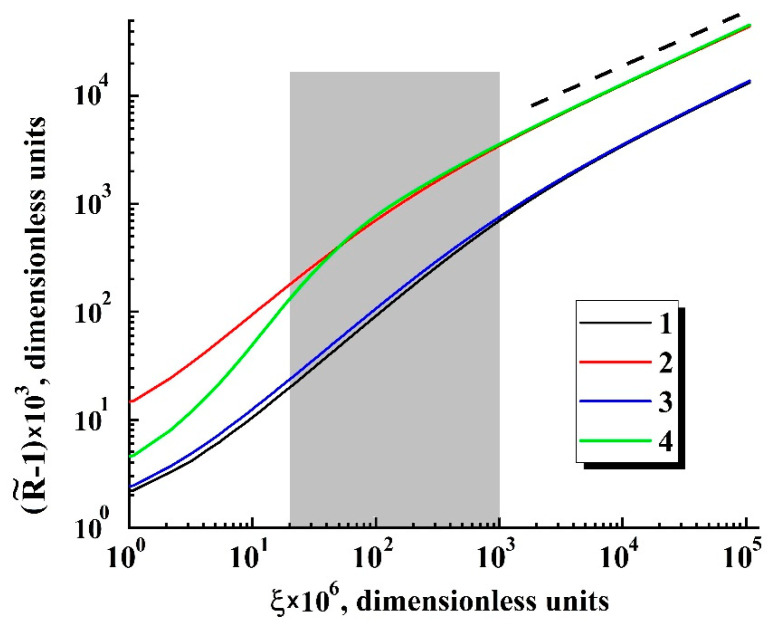
Expected variations of the normalized radii of embryos during the transient stage (results of modeling). T= 310 K; 1—$P_{\mathrm{int},0}=$ 4.0 MPa, Ξ= 0.001; 2—$P_{\mathrm{int},0}=$ 4.0 MPa, Ξ= 0.01; 3—$P_{\mathrm{int},0}=$ 8.3 MPa, Ξ= 0.001; 4—$P_{\mathrm{int},0}=$ 8.3 MPa, Ξ= 0.01. The dashed line displays a trend to the self-similar behavior.

Thus, the considered model predicts a self-similar character of depressurization-driven expansion of bubble embryos in an infinite viscous medium with the increasing dimensionless parameter ξ. Based on the general physical considerations, we can assume the existence of a transient regime between the moment of embryo nucleation and falling of the system “embryo-surrounding medium” into a self-similar expansion, which is controlled, in particular, by the current compressibility of the embryo-filling fluid (in our case, carbon dioxide). Based on the modeling results, we can conclude that this transient behavior occurs at the small detuning values ξ< 10^−3^ (and, accordingly, at a small temporal scale after the nucleation act). In particular, rough estimates of this scale give the value of the order of 10^−2^ to 10^−1^ s. Figure 9 displays an example of the modeled behavior of the expanding embryo in the transient mode for the expansion conditions sufficiently differing in the positions of the starting point at $\rho_{\mathrm{int}}=f_{T}\left(P_{\mathrm{int}}\right)$ isotherm. In accordance with applied experimental conditions, the 310 K isotherm of carbon dioxide was chosen for the comparative analysis because of remarkable variability of carbon dioxide compressibility along the isotherm line compared to higher temperatures. Consequently, the impact of fluid density variations on embryo expansion gradually vanishes in time and becomes negligible after the transient period. Following Taki [41], the transient stage (ξ< 10^−3^) is related to the dominating influence of environment viscosity on the embryo expansion, and the diffusion mass transfer to the embryo becomes crucial at larger time scales.

The grey-filled zone in Figure 9 marks the interval of ξ corresponding to the conditions of $\Delta R/\Delta P_{ext}$ evaluation from the recorded image sequences (see Section 2); position of the left boundary of the interval is defined by the temporal resolution of the used data acquisition system (33 ms) and applied depressurization rate. 

The width interval corresponds to the expected uncertainty in the evaluation $P_{ext,0}$ using the extrapolation procedure. The influence of Ξ on the embryo expansion dynamics in the interval of interest strongly dominates over the effect of isothermal compressibility. Based on the results of numerical analysis, we can accept with a reasonable accuracy the approximating relationship between $\Delta \tilde{R}/\Delta \xi$ and Ξ for further consideration:

(13) $$\frac{\Delta \tilde{R}}{\Delta \xi }\approx \chi \cdot \Xi^{\left(0.72\pm 0.08\right)}$$

where the dimensionless scaling factor χ is approximately equal to (1.00 ± 0.12) × 10^5^. With an increase in Δξ, the exponent in Equation (13) gradually decreases, approaching the value of 0.5 characteristic for a self-similar expansion mode.

## 4. Discussion of the Results

Taking into account Equations (12) and (13), the expansion rate of a traced individual embryo can be presented in the following form:

(14) $$\left|{\frac{\Delta R}{\Delta P_{ext}}|}_{R\to R_{c}}\right|\approx \left|\left(\frac{R_{c}}{P_{\mathrm{int},0}}\right)\cdot {\frac{\Delta \tilde{R}}{\Delta {\tilde{P}}_{ext}}|}_{R\to R_{c}}\right|\propto \left(\frac{R_{c}}{P_{\mathrm{int},0}}\right)\cdot \Xi^{0.72}\propto \frac{\eta^{0.72}D^{0.72}}{P_{\mathrm{int},0}^{1.72}R_{c}^{0.44}},$$

where the η, D, and $R_{c}$ parameters are considered as constant values at short time intervals between the nucleation act and evaluation of ${\Delta R/\Delta P_{ext}|}_{R\to R_{c}}$(see the aforementioned assumptions regarding the considered phenomenological model). Consequently, a significant increase in $\left|{\Delta R/\Delta P_{ext}|}_{R\to R_{c}}\right|$ with a decreasing value of $P_{ext,0}$ can be discussed in terms of the external pressure effect on the current values of these parameters. One of the crucial points in this discussion is the influence of $P_{ext,0}$ on the initial radius $R_{c}$ of a bubble embryo corresponding to the condition of its further stable expansion. We can use the following definition for the critical radius of a thermodynamically stable embryo in the solution [42]:

(15) $$R_{c}=\frac{2\Omega \sigma }{\Delta \mu }=\frac{2\Omega \sigma }{kT\psi }$$

where Ω is the volume per single molecule of the solute (carbon dioxide) in the bubble embryo, Δμ is a change in the chemical potential of nucleating species, and ψ is the dimensionless supersaturation factor. The term Ω directly relates to inverse current density of the solute in the embryo for the given internal pressure $P_{\mathrm{int},0}$. 

Depressurization of the “polylactide/carbon dioxide” system must lead to increasing the surface tension of bubble–polymer interfaces [39] and, presumably, to the decreasing supersaturation factor ψ. A combined action of these factors under isothermal conditions should lead to an increase in the critical radius $R_{c}$ of emerging bubble embryos with a decrease in external pressure. This assumption is particularly supported by the abovementioned results relating a reduction in the empirical dependencies R=f(t) (Figure 5a) to the self-similar function $\tilde{R}=\psi \left\{\tilde{\xi }\right\}$ (Figure 8a systematic increase in the fitting values $R_{c}$ with a decreasing external pressure corresponding to emergence of a stable embryo growth). Assuming the linear mode of CO_2_ diffusion in the plasticized polylactide in a wide range of external pressures, and taking into account a relatively narrow interval of constant temperatures applied in depressurization experiments (from 310 to 338 K), we can assume a rather insignificant influence of the diffusion coefficient D on the Ξ parameter compared to other factors ($R_{c}$, $P_{\mathrm{int},0}$, and η). Taking into account a competitive influence of these parameters on Ξ, we can assume rather modest changes of Ξ within the examined range of external pressures and temperatures. This assumption is particularly supported by the results of the experimental data fitting based on the obtained dependence $\tilde{R}=\psi \left(\tilde{\xi }\right)$ (Figure 8, the adjustable value of Ξ varies from ≈1.8 × 10^−3^ to ≈2.9 × 10^−3^ with a simultaneous approximate three-fold increase in $R_{c}$). Moreover, the overall variations of the adjustable parameters $R_{c}$ and Ξ for the analyzed experimental datasets (Figure 5a) reasonably agree with the established power-law trend $\left|{\Delta \bar{R}/\Delta {\bar{P}}_{ext}|}_{R\to R_{c}}\right|\propto {\left({\bar{P}}_{ext,0}\right)}^{-2.7}$ (Figure 5b).

**Figure 10:**
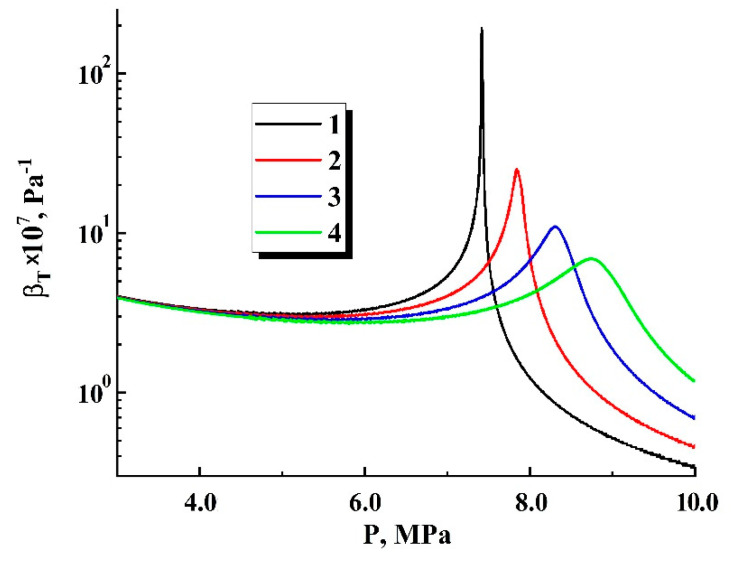
Dependencies of isothermal compressibility of carbon dioxide on the pressure (recovered using isothermal data published elsewhere [40]). 1—T=304.4 K (near the critical temperature); 2—T= 307.0 K; 3—T= 310.0 K; 4—T= 313.0 K.

**Figure 11:**
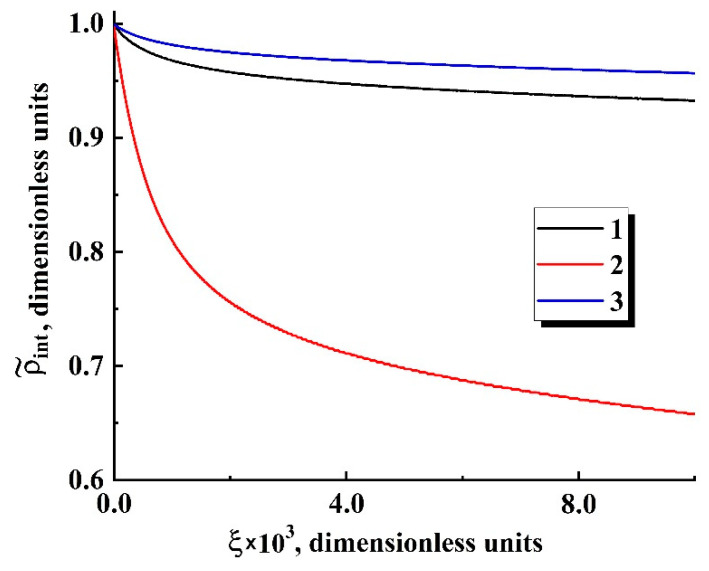
Jumps of the normalized carbon dioxide density in expanding embryos during the transient stage (results of modeling). T= 310 K; Ξ= 0.001. 1—$P_{\mathrm{int},0}=$ 4.0 MPa; 2—$P_{\mathrm{int},0}=$ 8.3 MPa; 3—$P_{\mathrm{int},0}=$ 10.0 MPa.

The experimentally observed absence of stably expanding embryos in the region of ${\bar{P}}_{ext,0}$ around the critical pressure $P_{c}\approx$ 7.38 × 10^6^ Pa (a gap between ${\bar{P}}_{ext,0}\approx$ 6.6 × 10^6^ Pa and ${\bar{P}}_{ext,0}\approx$ 8.3 × 10^6^ Pa in Figure 5b) can be interpreted in terms of a joint influence of two factors in the course of the transient stage. This stage is characterized by a rapid decrease in the fluid density in the embryos, whereas the relevant factors are quasi-adiabatic cooling of a rapidly expanding fluid and an abrupt increase in its compressibility near the critical point. Figure 10 illustrates a major increase in the isothermal compressibility $\beta_{T}={\left\{\left(1/\rho \right)\cdot \left(\partial \rho /\partial P\right)\right\}}_{T}$ of carbon dioxide when approaching the critical point; the curves were obtained on the basis of the CO_2_ isothermal data generated using the NIST calculator of thermophysical properties of fluid systems [40]. High fluid compressibility inside the expanding embryo causes its significant density decrement during the transient stage; for example, Figure 11 displays the initial fragments of ${\tilde{\rho }}_{\mathrm{int}}\left(\xi \right)$ dependencies calculated using the considered model. Curves 1 and 2 correspond to the values of $P_{ext,0}$ far enough from the critical pressure (4.0 × 10^6^ Pa and 1.0 × 10^7^ Pa, respectively), whereas curve 3 corresponds to the transient mode of expansion in the case of the relatively small $P_{ext,0}$ detuning from the critical pressure ($P_{ext,0}=$8.3 × 10^6^ Pa). The modeled data correspond to T= 310 K as the minimal temperature examined in our experiments; at the same time, it is characterized by the maximal compressibility of carbon dioxide near the critical pressure (Figure 10). All theoretical dependencies were obtained under assumptions of a relatively low diffusion income to the mass of fluid in the embryo during the transient stage (Ξ= 0.001) and isothermality of the expanding embryo.

**Figure 12:**
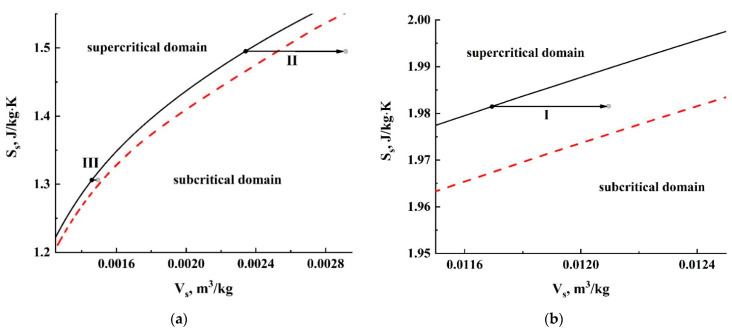
Expected effect of quasi-adiabatic cooling of carbon dioxide in the expanding embryos during the transient stage. Changes in the thermodynamic state of CO2 are presented in the “specific volume - specific entropy” coordinate system. Figure (**a**) corresponds to lower values of *V_s_* and *S_s_* (higher initial pressures, transitions II and III), while figure (**b**) displays transition I occurring at a lower initial pressure. The considered cases correspond to those displayed in Figure 11. (*I→1, II→2, III→3*). The red dashed line (T= 304.4 K) defines a borderline between supercritical and subcritical domains. The black solid line (T= 310 K) displays the dependence of the specific entropy on the specific volume of carbon dioxide.

It should be noted that isothermality assumptions at the transient stage are rather debatable in terms of the exact quantification of current embryo parameters (primarily due to a possible cooling of the fluid in the embryo) because, in certain cases, it is characterized by large density decrements. However, a qualitative examination of the physical mechanisms leading to appearance of the abovementioned gap in the “pressure-expansion rate” diagram near the critical pressure can be carried out using this simplifying assumption. A quasi-adiabatic cooling of carbon dioxide during the transient stage of embryo expansion can be qualitatively analyzed in terms of a “specific volume ($V_{sp}=\rho^{-1}$)-specific entropy ($S_{sp}$)” diagram for carbon dioxide. Assuming a negligible heat and mass exchange between the expanding embryo and surrounding polymer in the course of the transient stage due to its short duration, we can state quasi-isentropicity of the expanding fluid in the embryo during the transient stage. As an example, Figure 12 displays the expected effect of quasi-adiabatic expansion of carbon dioxide for the cases presented in Figure 11. The dashed curve corresponds to the temperature T= 304.4 K, slightly exceeding the critical temperature of carbon dioxide ($T_{c}\approx$304.13 K), and the solid curve displays the $S_{sp}\left(V_{sp}\right)$ dependence at T= 310 K. The corresponding datasets were obtained using the online NIST calculator [40]. The black arrows display quasi-isentropic transitions between the initial and final states of carbon dioxide during the transient stage; their dimensions are determined by the relative changes in the specific volume, which are correlated with the density decrements during the transient stage. 

Accordingly, transitions (1) and (3) corresponding to relatively small values of fluid compressibility are characterized by a relatively subtle cooling effect; the fluid remains in a supercritical state. On the contrary, in the case of high compressibility (2), there is a dramatic change in the fluid state with sufficiently deep cooling and transition to the subcritical region. In turn, this should lead to formation of a two-phase system “liquid carbon dioxide-gaseous carbon dioxide” in the embryo, loss of thermodynamic stability and, finally, embryo collapse.

Considering the presented results of the semi-quantitative analysis (Figure 10, Figure 11 and Figure 12), we can assume that at sufficiently large variations in the fluid density at the intermediate stage, the discussed effect can cause a loss of thermodynamic stability with a subsequent collapse of the embryo. Moreover, quasi-adiabatic cooling at appropriately large density decrements can act as a self-sustaining avalanche-like process (the cooling leads to an increasing compressibility; in turn, this causes a deeper cooling, etc.). Accordingly, at the qualitative level, such mechanisms presumably explain the existence of the “zero-rate” gap in the “pressure-rate” diagram (Figure 5b).

It is important to compare duration of the transient stage with the characteristic time $\tau_{th}$ of thermal relaxation for the considered system; this characteristic time can be roughly estimated as $\tau_{th}\approx R_{c}^{2}/a_{CO_{2}}$, where $a_{CO_{2}}$ is the thermal diffusivity of carbon dioxide. In turn, the thermal diffusivity is defined as $\lambda_{CO_{2}}/\rho_{CO_{2}}c_{p,CO_{2}}$, where $\lambda_{CO_{2}},\rho_{CO_{2}},c_{p,CO_{2}}$ are the thermal conductivity, density, and specific thermal capacity, respectively (they can be evaluated using the online NIST calculator [40]). Considering these carbon dioxide parameters for the range of examined pressures and temperatures, we obtain the expected range of $a_{CO_{2}}$ as ≈10^−9^ to 10^−7^ m^2^/s (minimal values occur near the critical point due an abrupt increase in specific thermal capacity). On the other hand, a characteristic size of the embryo at the transient stage is of the order of several micrometers; accordingly, the characteristic time of thermal relaxation does not exceed a few tenths of a second and is comparable to duration of the transient stage. This allows us to conclude that the self-similar stage of embryo expansion occurs under condition of its thermal equilibrium with the environment.

This analysis refers to an intermediate stage of polymer foaming between the nucleation due to thermally induced density fluctuations at the molecular level and expansion of an ensemble of close-packed bubbles. In addition to the depressurization-governed expansion rate of steadily growing bubble embryos, the nucleation rate is also a key factor controlling the structural properties of the synthesized polymer foam. The influence of quasi-isothermal depressurization conditions on the nucleation rate in the “carbon dioxide-polymer” system was previously discussed [25,43,44] in terms of the system evolution along certain trajectories on the “solute concentration-pressure” phase diagram. A crucial factor controlling nucleation kinetics is the current value of the Gibbs energy of nucleus formation depending on the position of the system with respect to binodal and spinodal lines for the examined system at the given temperature. The probability of nucleation with a further stable growth of the bubble embryo is equal to 0 if the system evolves in the region of the phase diagram above the binodal line. Additionally, the system trajectories below the spinodal line correspond to the spinodal decomposition mode, when the stable growth of bubble embryos is characterized by a near-to-zero probability. Favorable conditions are achieved only when the trajectories are located in the diagram zone between bimodal and spinodal lines. Additionally, the trajectory characterized by the lower average Gibbs energy along it is more preferable due to a higher nucleation rate and smaller differences between the rates of heterogeneous and homogeneous nucleation [25]. In combination with a low expansion rate of bubble embryos, this feature leads to a more homogeneous structure of the synthesized polylactide foam, as clearly seen in Figure 1ii.

Finally, we should state that one of the main assumptions of the considered model, namely, the spherical shape of stably expanding bubble embryos, is fulfilled under certain limiting conditions. These limitations relate to the absence of influence of the environment (neighboring embryos, container surface, etc.) on the development of a single embryo, and isotropic and nearly uniform macroscopic distribution of the pressure field in the polymer matrix. This is valid for experimental conditions used in our case (an early stage of pre-foaming, and selection of the tracked embryo far enough from the container boundaries, see Figure 3). However, at later stages of pre-foaming and during transition to intensive expansion of the foam, going beyond these limitations leads to strong deviations in the shape of the developing pores from the regular spherical shape (see, for example, Figure 1i). Other factors leading to significant deviations of the pore shapes from the spherical shape are pressure field anisotropy and anisotropic mass transfer during the evolution of the pore ensemble. In particular, the influence of these factors on the pore shapes and orientations manifests itself during application of the foam injection molding combined with batch foaming [22].

## 5. Conclusions

We can conclude that expansion of bubble embryos during slow quasi-isothermal depressurization of the “polylactide-carbon dioxide” system, which precedes expansive growth of the polymer foam, can be adequately described in terms of self-similarity using a small number of dimensionless variables. These variables are the dimensionless time lapse and depressurization rate, and expansion dynamics are controlled by the dimensionless factor Ξ which depends on viscosity of the plasticized polymer, CO_2_ diffusion coefficient in the polymer, initial radius of an embryo, and initial internal pressure in the embryo.

The obtained experimental data demonstrate a strong inverse dependence of the expansion rate on the current pressure. Thus, as the current pressure decreases from 10 MPa to 3.5 MPa, the expansion rate increases from ≈0.1 to ≈10 µm/s. This dependence can be approximated by the power function with an exponent equal to ≈2.7. The obtained value of an exponent satisfactorily agrees with the expected changes in the system parameters within the examined ranges of pressure and temperature.

With exception of a short-term transient stage, the dynamics of self-similar expansion are independent of the thermo-physical properties of the fluid filling the embryo. The expected duration of this transient stage is at least several tens of milliseconds and is comparable to the time of capturing several single frames in the analyzed video streams. Accordingly, the analyzed self-similar expansion of embryos is an aftereffect of this transient process. On the other hand, remarkable transient variations in the fluid density inside the embryos should lead to their short-term thermodynamic non-equilibrium, and in certain cases can cause their collapse. This feature presumably manifests itself in existence of the “dead zone” around the CO_2_ critical pressure.

Despite some simplifying assumptions, the considered model adequately predicts observed features of embryo expansion starting from finalization of the transient stage and ending with sufficiently large values of the normalized pressure drop. The resulting structure of the polymer foam, in addition to the average expansion rate of individual embryos, is also determined by the nucleation rate. Therefore, the foaming modes providing low expansion rates in combination with high nucleation rates are preferable in terms of formation of polymer foams with a more uniform structure.

In our opinion, the obtained results may be of interest not only for improving the polymer foaming technology, but also for a further insight into the physical aspects of the bubbling dynamics.

## Figures and Tables

**Table 1:** The experimental conditions for the datasets 1–6 (Figure 4, Figure 5a).

Number of the Dataset	$P_{ext}^{in},\;\mathrm{MPa}$	T **, K**
1	8.0	338
2	8.0	323
3	8.0	338
4	14.5	338
5	11.5	323
6	11.5	310

**Table 2:** The experimental conditions for the datasets 1–9 (Figure 5b).

Number of the Dataset	$P_{ext}^{in},\;\mathrm{MPa}$	T **, K**
1	11.5	310
2	7.0	323
3	11.5	323
4	14.5	333
5	7.0	310
6	14.5	338
7	14.5	323
8	8.0	323
9	8.0	338

**Table 3:** The fitting parameters ($R_{c},\Xi$) for the datasets 1–6 (Figure 8).

Number of the Dataset	$R_{c},\;µm$	Ξ
1	10.1 ± 2.5	(2.9 ± 0.6) × 10^−3^
2	13.5 ± 3.2	(2.8 ± 0.6) × 10^−3^
3	13.8 ± 3.3	(2.9 ± 0.6) × 10^−3^
4	4.5 ± 1.0	(1.8 ± 0.4) × 10^−3^
5	6.0 ± 1.5	(2.0 ± 0.6) × 10^−3^
6	8.0 ± 1.9	(2.2 ± 0.8) × 10^−3^

## Data Availability

The data presented in this study are available on request from the corresponding author.
